# RETRACTION: Effect of passive versus active abdominal drainage on wound infection after pancreatectomy: A meta‐analysis

**DOI:** 10.1111/iwj.70113

**Published:** 2024-10-16

## Abstract

Retraction: HanY. , WuZ. , SongJ. , ZhangQ. , WeiL. , and LuH. , “Effect of Passive Versus Active Abdominal Drainage on Wound Infection after Pancreatectomy: A Meta‐Analysis,” International Wound Journal 21, no. 3 (2024): e14755, 10.1111/iwj.14755.38453160 PMC10920029

The above article, published online on 07 March 2024, in Wiley Online Library (http://onlinelibrary.wiley.com/), has been retracted by agreement between the journal Editor in Chief, Professor Keith Harding; and John Wiley & Sons, Ltd. It came to the publisher's attention from a third party that a number of articles shared concerning similarities in format and structure. Following an investigation by the publisher, the retraction has been agreed on as the peer review and publishing process for this article were found to be manipulated. The authors did not respond to the notice of retraction.

Retraction: Y. Han , Z. Wu , J. Song , Q. Zhang , L. Wei , and H. Lu , “Effect of Passive Versus Active Abdominal Drainage on Wound Infection after Pancreatectomy: A Meta‐Analysis,” International Wound Journal 21, no. 3 (2024): e14755, 10.1111/iwj.14755.38453160 PMC10920029

The above article, published online on 07 March 2024, in Wiley Online Library (http://onlinelibrary.wiley.com/), has been retracted by agreement between the journal Editor in Chief, Professor Keith Harding; and John Wiley & Sons, Ltd. It came to the publisher's attention from a third party that a number of articles shared concerning similarities in format and structure. Following an investigation by the publisher, the retraction has been agreed on as the peer review and publishing process for this article were found to be manipulated. The authors did not respond to the notice of retraction.

